# Omenn Syndrome: inflammation and autoimmunity

**DOI:** 10.1186/1479-5876-9-S2-I5

**Published:** 2011-11-23

**Authors:** Anna Villa

**Affiliations:** 1 Milan Unit, Istituto di RicercaGenetica e Biomedica, ConsiglioNazionaledelleRicerche, Milan, Italy; 2 Istituto ClinicoHumanitas, Rozzano, Milan, Italy

## Background

Omenn Syndrome (OS) is a rare inherited disorder presenting with early-onset generalized erythrodermia, alopecia, chronic diarrhea, lymphoadenopathy, failure to thrive, and recurrent infections. The immunological phenotype is characterized by eosinophilia, virtual absent circulating B cells but elevated IgE serum levels. T cells are normal or increased and display a highly restricted, oligoclonal TCR repertoire. Because of overwhelming infections and loss of proteins, the disease is fatal, unless treated with allogeneic bone marrow transplantation, which is variably successful. We have previously demonstrated that mutations in either RAG1 and RAG2, impairing, but not abolishing, the first steps of V(D)J recombination, are the main cause of this syndrome [1]. Nonetheless, a compelling explanation for the autoimmune features of OS has proven elusive. The pathogenesis is likely multifactorial. We have shown that the reduced thymopoiesis in OS patients is accompanied by defects in thymic epithelial cell maturation and/or homeostasis, impaired expression of autoimmune regulator (aire) protein and poor generation of thymic CD4+CD25+Foxp3+ natural regulatory T cells [2,3]. While these observations emphasize the potential role of T cell tolerance impairments in sustaining the immune dysregulation of OS, a possible contribution of defects in other immune cells, such as antigen presenting cells, has not been investigated. In addition, numerous questions remain surrounding the disease pathogenesis. To gain insights into the mechanisms underlying autoimmunity, we have recently generated a murine model (the *Rag2^R229Q/R229Q^*mouse) carrying in Rag2 gene a mutation responsible for OS in many patients. This mouse model recapitulates the phenotype and many of the clinical manifestations of OS patients [4]. We have used this model to further investigate T and B cell contribution in the pathogenesis of autoimmune manifestations in OS.

## Results

To characterize B cell defects and their contribution to the disease, we studied the hypomorphic R229Q rag2 mice. Consequent to a developmental arrest at the early pro-B stage, peripheral B cell pool in *Rag2^R229Q/R229Q^*mouse showed a strong reduction in size and an altered composition. The majority of B cells retained an immature phenotype, while a few IgM^+^ B cells displayed higher expression of activation markers. Nonetheless, a normal or even increased compartment of immunoglobulin secreting cells is generated. The exaggerated activation and plasmacytic differentiation of *rag2^R229Q^* B cells correlated to elevated levels of homeostatic cytokines, leading to increased Blimp-1 and Xbp-1 expression. Moreover, analysis of serum autoantibodies revealed the presence of anti-dsDNA, suggestive of defaults in B-cell selection and tolerance. Collectively, our findings for the first time point out a role for B cells in OS pathology [5].

## Conclusions

In conclusion, we show that in OS persistent inflammation favours the expansion and the exaggerated plasmacytic differentiation of an oligoclonal B cell population with autoreactive potential. Thus, we propose that breakdown in B cell selection and tolerance contributes to the development of autoimmunity and atopy in this disease.
